# No Impact of Fluconazole to Echinocandins Replacement as First-Line Therapy on the Epidemiology of Yeast Fungemia (Hospital-Driven Active Surveillance, 2004–2017, Paris, France)

**DOI:** 10.3389/fmed.2021.641965

**Published:** 2021-04-20

**Authors:** Stéphane Bretagne, Marie Desnos-Ollivier, Karine Sitbon, Olivier Lortholary, Didier Che, Françoise Dromer, Participants of the YEASTS

**Affiliations:** ^1^Institut Pasteur, CNRS, Unité de Mycologie Moléculaire, Centre National de Référence Mycoses Invasives et Antifongiques, Paris, France; ^2^Laboratoire de Parasitologie-Mycologie, Hôpital Saint Louis, AP-HP, Paris, France; ^3^Université de Paris, Paris, France; ^4^Service des Maladies Infectieuses et Tropicales, Centre d'Infectiologie Necker-Pasteur, Hôpital Necker-Enfants Malades, APHP, IHU Imagine, Paris, France; ^5^Santé publique France, Saint Maurice, France

**Keywords:** fungemia, candidemia, epidemiology, hematological malignancy, solid cancer, intensive care unit, echinocandins, fluconazole

## Abstract

Replacement of fluconazole by echinocandins as the first-line therapy for yeast-related fungemia could have an impact on both the mortality rate and the epidemiology of yeast species responsible for candidemia. We analyzed the individual clinical and microbiological data collected through the active surveillance program on yeast fungemia (YEASTS program, 2004–2016, Paris area, France) within 14 University Hospitals. The cohort included 3,092 patients [male:female ratio: 1.56; median age 61.0 years (IQR: 23.8)]. The mean mortality rate within 30 days was 38.5% (1,103/2,868) and significantly higher in intensive care units (690/1,358, 50.8%) than outside (413/1,510, 27.4%, *p* < 0.0001) without significant change over time. The yeast species distribution [*Candida albicans* (*n* = 1,614, 48.0%), *Candida glabrata* (*n* = 607, 18.1%), *Candida parapsilosis* (*n* = 390, 11.6%), *Candida tropicalis* (*n* = 299, 8.9%), *Candida krusei* (*n* = 96, 2.9%), rare species (*n* = 357, 10.6%)], minimal inhibitory concentration distribution, and the distribution between the patient populations (hematological malignancies, solid tumors, without malignancy) did not change either while the proportion of patients ≥60-years increased from 48.7% (91/187) in 2004 to 56.8% (133/234) in 2017 (*p* = 0.0002). Fluconazole as first-line therapy dramatically decreased (64.4% in 2004 to 27.7% in 2017, *p* < 0.0001) with a corresponding increase in echinocandins (11.6% in 2004 to 57.8% in 2017, *p* < 0.0001). Survival rates did not differ according to the first antifungal therapy. The progressive replacement of fluconazole by echinocandins as the first-line antifungal therapy was not associated with change in global mortality, regardless of species involved and antifungal susceptibility profiles. Other factors remain to be uncovered to improve the prognosis of yeast fungemia.

## Introduction

Candidiasis is associated with mortality as high as 40%, acknowledging that patients with this condition usually present several entangled risk factors impacting the prognosis (1). To improve the prognosis and to harmonize diagnostic and therapeutic strategies, several guidelines have been released worldwide for different patient populations (2–10). One of the recommendations is the strong incentive for using echinocandin instead of fluconazole as first-line therapy based on analysis of randomized trials (11, 12). One the reasons usually advocated is the limited spectrum of activity of fluconazole and the resistance or decreased susceptibility to fluconazole of some *Candida* species. Indeed, the possible shift toward non-*albicans Candida* species and the emergence of resistance to antifungal drugs pose a serious potential health threat considering the poor prognosis of candidiasis and the limited number of systemic antifungal drugs available (1, 13). It is therefore of utmost interest to evaluate the impact of such therapeutic recommendations on both the epidemiology of the different species and the associated mortality rate.

To measure the burden of candidiasis, an active surveillance program on yeast fungemia within university hospitals in the Paris area was set up by the French National Reference Center of Invasive Mycoses and Antifungals (NRCMA). This surveillance includes all the episodes of yeast fungemia, and the centralization of all isolates by the NRCMA allowing for standardized characterization of the fungal species and their susceptibility to antifungal drugs. We describe here the epidemiology of yeast-associated fungemia, mainly candidemia, and the trends in mortality in correlation with individual treatment over 14 years in the Paris area.

## Materials and Methods

### Yeasts Program

The YEASTS program is an active surveillance program on yeast fungemia described in details elsewhere (14, 15). The current analysis concerns all episodes of yeast fungemia (excluding only *Cryptococcus neoformans*) diagnosed in all ages, notified by the 14 university hospitals which sustainably participated from January 2004 to December 2017. All sent the corresponding yeast isolates to the NRCMA where the species identification was checked and antifungal susceptibility determination performed using European Committee on Antimicrobial Susceptibility Testing (EUCAST) methodology as described (14, 15). In parallel, clinical data, treatment and outcome were reported through a secure website.

The day of blood sampling was considered as the date of fungemia. An incident case corresponded to the first episode of positive blood culture. A recurrent episode was considered in case of isolation of the same species at least 10 days after the initial isolation or of a new species with no time limit. Both single (one species) and mixed (>1 species) episodes were considered. Patients were grouped into three categories (hematological malignancy hereafter “Hematology”), solid tumor (any kind of solid tumor at the time of sampling, hereafter “Oncology”) or no malignancy (all other cases, hereafter “No-malignancy”). Cases were classified regarding their occurrence during a stay in intensive care unit (ICU) or not. All isolates were considered for the analysis of species distribution overtime, and all episodes were analyzed for the impact of antifungal prescription (prior exposure or therapeutic), but only incident episodes were considered for the rest of the analysis to avoid autocorrelation.

Initial (first 48 h) prescription of antifungal treatment and outcome within 30 days after sampling were recorded with the notion of antifungal prior exposure, whatever the drug, dosage and duration, within 30 days before fungemia. Mortality regarding first-line therapy was analyzed for patients with available outcome and only if the positivity of blood culture was known before death. Initial prescription was divided in four groups: fluconazole alone, echinocandin alone (aggregating caspofungin and micafungin), other antifungal treatments (liposomal amphotericin B, voriconazole, posaconazole, itraconazole and drug combinations), and no treatment, providing that the patient was still alive at the time of fungemia diagnosis and that treatment were prescribed for at least 48 h before death eventually occurred.

Isolates with decreased susceptibility to fluconazole were those intrinsically resistant (*Candida krusei*) or those tested with fluconazole minimum inhibitory concentration (MIC) >32 mg/L according to EUCAST Antifungal Agents Breakpoint tables for interpretation of MICs (https://www.eucast.org/fileadmin/src/media/PDFs/EUCAST_files/AFST/Clinical_breakpoints/Antifungal_breakpoints_v_9.0_180212.pdf), while those with decreased susceptibility to caspofungin were those intrinsically resistant (basidiomycetes and *Geotrichum* spp.) or those tested with a caspofungin MIC >0.25 mg/L (16).

Incidence rates of the 14 participant hospitals were calculated per 1,000 admissions and 10,000 hospitalization days using annual hospital activity data for the years 2010–2017, because of a change in 2009 preventing use of the earlier years (Statistique annuelle des établissements de Santé, French Ministry of Health: https://www.sae-diffusion.sante.gouv.fr/sae-diffusion/recherche.htm). Univariate analysis was based on Chi2 or Fisher's exact-test for discrete variables. Chi2-test for trends was used to determine trends over time. Survivals were determined by Kaplan-Meier analysis and compared by the log rank-test. Data were analyzed using Stata® Software (version 15.0; College Station, TX).

### Ethics

The research described herein was carried out in compliance with the French law and the Declaration of Helsinki (as adopted in 2000), and was approved by the Institut Pasteur institutional review board (IRB #2009-34) and the “Commission Nationale de l'Informatique et des Libertés” according to French regulation.

## Results

### Global Epidemiology

Over the 14 years of the survey, 3,257 episodes of yeast fungemia were recorded including 165 recurrences [median onset (IQR) = 23 [67] days after incident episode], and 97 episodes with more than one species. Oncology patients experienced less recurrent episodes of fungemia (37/1,025, 3.6%) than did hematology patients (39/657, 5.9%) or patients with no malignancy (89/1,575, 5.5%) (*p* = 0.036). Overall, 3,092 patients were concerned including 136 children <15-year-old. The male: female ratio was 1.6 (not different in adults and children <15-year-old) and did not change over time (*p* = 0.7966). The median age was 61.0 years (IQR: 23.8) with a significant increase over time. While the proportion of children remained stable (4.4%), that of patients over 60-year of age increased from 48.7% (91/187) in 2004 to 56.8% (133/234) in 2017 (*p* = 0.0002) and that of patients over 80-year of age increased from 9.1% (17/187) in 2004 to 15.4% (36/234) in 2017 (*p* = 0.039).

The population was composed of patients with malignancy (*n* = 1,606, i.e., 51.9%) including 618 (20.0%) Hematology patients, 988 (31.9%) Oncology patients, and 1,486 (48.1%) No-malignancy patients (Table 1). No significant variation in the proportion of the three different groups was observed over time (data not shown). Almost half of the patients (1,444/3,092; 46.7%) were in ICU when fungemia was diagnosed. In ICU, most of the patients had no malignancy (950/1,444; 65.8%) while Oncology patients represented the first group outside ICU (690/1,648; 41.9%). In the Hematology group only the proportion of stay in ICU significantly increased over time when considering incident episodes of yeast fungemia (from 24.5 to 34.0%, *p* = 0.0002) or all episodes (not shown). Central venous catheters were reported in the majority of episodes (2,324/3,257; 71.4%), more often in ICU (1,161/1,526; 76.1%) than outside ICU (1,163/1,731; 67.2%; *p* < 0.0001), and more often in the Hematology (561/657 85.4%) and Oncology (788/1,025; 76.9%) than in the No-malignancy (975/1,575; 61.9%) groups (*p* < 0.0001) with no significant change over time. Considering the 14 centers, the incidence rate/1,000 admissions increased from 0.63 to 0.77 (2010–2017). The incidence rate/10,000 hospitalization day increased from 1.12 to 1.26 (2010–2017). In the same period, the number of beds decreased by 5.6% (from 7,033 to 6,638).

**Table 1:** Characteristics of the 3,092 patients who experienced a first episode of yeast fungemia (Paris, YEASTS program, 2004–2017).

	**Total (*n*= 3,092)**	**ICU (*n* = 1,444)**	**No ICU (*n* = 1,648)**	***p***
**Median age (IQR)**	**61.0 (23.8)**	**61.2 (22.6)**	**60.8 (24.9)**	0.702
**Sex ratio (M:F)**	**1.56:1**	**1.66:1**	**1.47:1**	0.095
**Hematology**, ***n*** **(%)**	**618 (20.0%)**	**196 (13.6%)**	**422 (25.6%)**	<0.0001
Lymphoma	225 (36.4%)	78 (39.8%)	147 (34.8%)	0.107
Acute leukemia	249 (40.3%)	67 (34.2%)	182 (43.1%)	
Other	144 (23.3%)	51 (26.0%	93 (22.0%)	
**Oncology**, ***n*** **(%)**	**988 (32.0%)**	**298 (20.6%)**	**690 (41.9%)**	0.823
Digestive tract	472 (47.8%)	138 (46.3%)	334 (48.4%)	<0.0001
Genital tract	112 (11.3%)	22 (7.4%)	90 (113.0%)	
Urinary tract	105 (10.6%)	39 (13.1%)	66 (9.6%)	
ENT	78 (7.9%)	22 (7.4%)	56 (8.1%)	
Diverse	221 (22.4%)	77 (25.8%)	144 (20.9%)	
**No malignancy**, ***n*** **(%)**	**1,486 (48.0%)**	**950 (65.8%)**	**536 (32.5%)**	<0.0001
**Recent surgery**, ***n*** **(%)**	**560 (37.7%)**	**352 (37.0%)**	**208 (38.8%)**	0.787
Digestive tract	244 (43.6%)	167 (47.4%)	77 (37.0%)	<0.0001
Urinary tract	29 (5.2%)	9 (2.6%)	20 (9.6%)	
Heart + vascular	113 (20.2%)	89 (25.3%)	24 (11.5%)	
Orthopedic	108 (19.3%)	47 (13.4%)	61 (29.3%)	
Diverse	66 (11.8%)	40 (11.4%)	26 (12.5%)	
**Organ transplantation**, ***n*** **(%)**	**149 (10.0%)**	**104 (11.0%)**	**45 (8.4%)**	0.116
Kidney	42 (28.2%)	22 (21.2%)	20 (44.4%)	0.001
Liver	77 (51.7%)	53 (51.0%)	24 (53.3%)	
Heart	15 (10.1%)	156 (14.4%)	-	
Other	15 (10.1%)	14 (13.5%)	1 (2.2%)	
**Bacterial infection**, ***n*** **(%)**	**331 (22.3%)**	**231 (24.3%)**	**100 (18.7%)**	0.012
**HIV infection**, ***n*** **(%)**	**29 (2.0%)**	**16 (1.7%)**	**13 (2.4%)**	0.321
**Intravenous drug addiction**, ***n*** **(%)**	**19 (1.3%)**	**7 (0.7%)**	**12 (2.2%)**	0.013
**Corticosteroid therapy**, ***n*** **(%)**	**38 (2.6%)**	**21 (2.2%)**	**17 (3.2%)**	0.260
**Severe burns**, ***n*** **(%)**	**26 (1.8%)**	**26 (2.7%)**	**-**	<0.0001
**Central venous catheter as the only reported risk factor**, ***n*** (%)^*^	**197 (13.3%)**	**144 (15.2%)**	**53 (9.9%)**	0.004
**No reported risk factor**, ***n*** **(%)**	**176 (11.8%)**	**132 (13.9%)**	**44 (8.2%)**	0.001

* *Including other foreign devices*.

### Trends in Mortality

**Figure 1:**
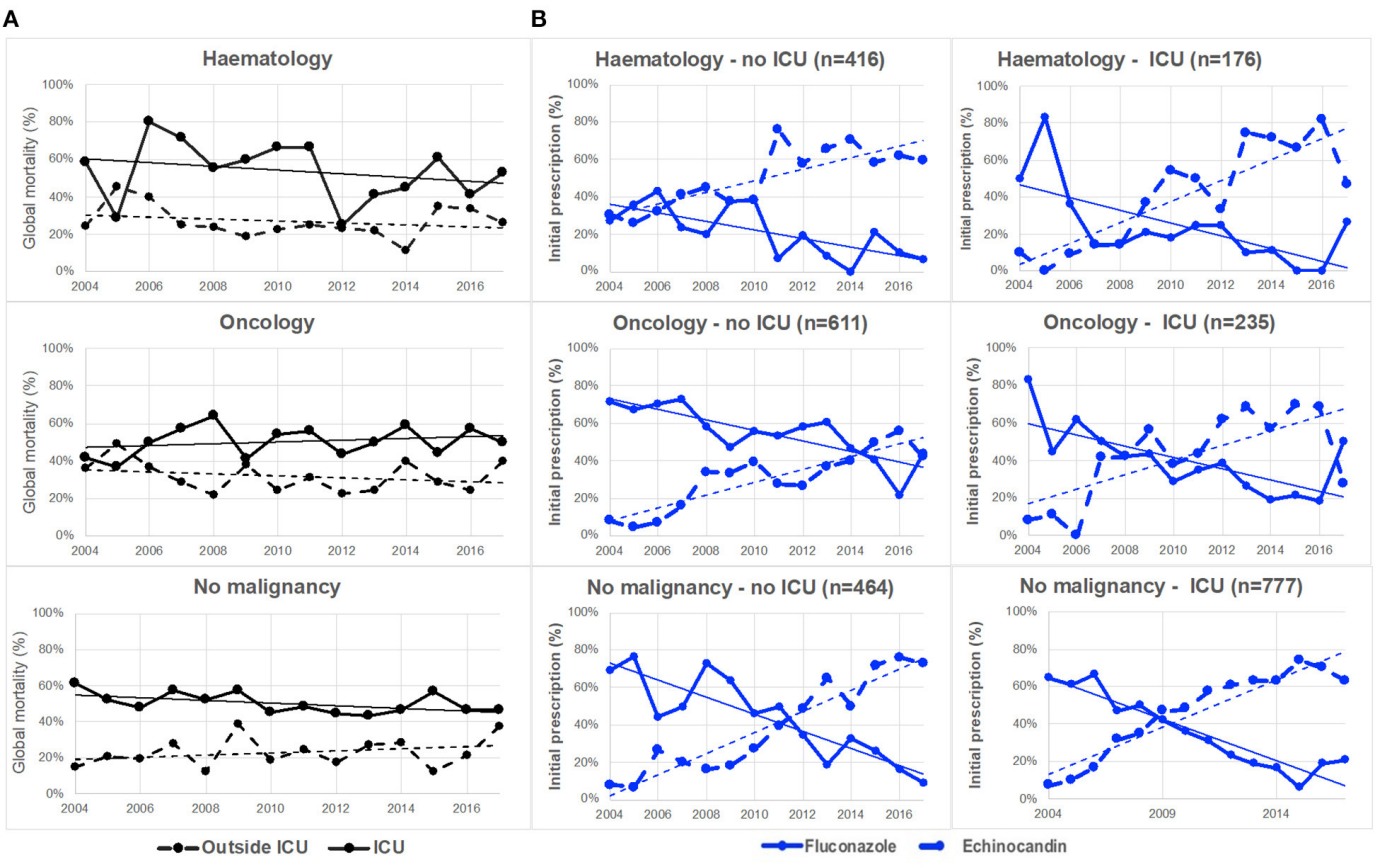
Evolution of global mortality and first-line antifungal drug prescription in patients with hematological malignancy, oncology or no malignancy, inside or outside intensive care unit (ICU) (YEASTS program, Paris area, 2004–2017). **(A)** Global mortality did not change significantly over time inside (solid black line) and outside (dotted black line) ICU in the various categories of patients. **(B)** The evolution of fluconazole (solid blue line) and echinocandins (dotted blue line) prescription significantly (*p* < 0.001) differed inside and outside ICU in the three populations of patients. The switch from fluconazole to echinocandins as first-line therapy did not occur at the same date in the six populations studied.

The outcome was available for 2,868 patients and 1,103 (38.5%) died within 30 days of diagnosis of candidemia. Of note, 326 of them (30.7%) died before diagnosis was made. The global mortality was significantly higher in ICU (690/1,358, 50.8%) than outside (413/1,510, 27.4%, *p* < 0.0001), but it did not change significantly over the study period (69/180; 38.3% in 2004 to 86/207; 41.6% in 2017, *p* = 0.221). There was no trend whatever the environment considered (ICU or no ICU) and the population studied (Hematology, Oncology or No-malignancy) (Figure 1A).

### Trends in Species Recovered and MICs

**Figure 2:**
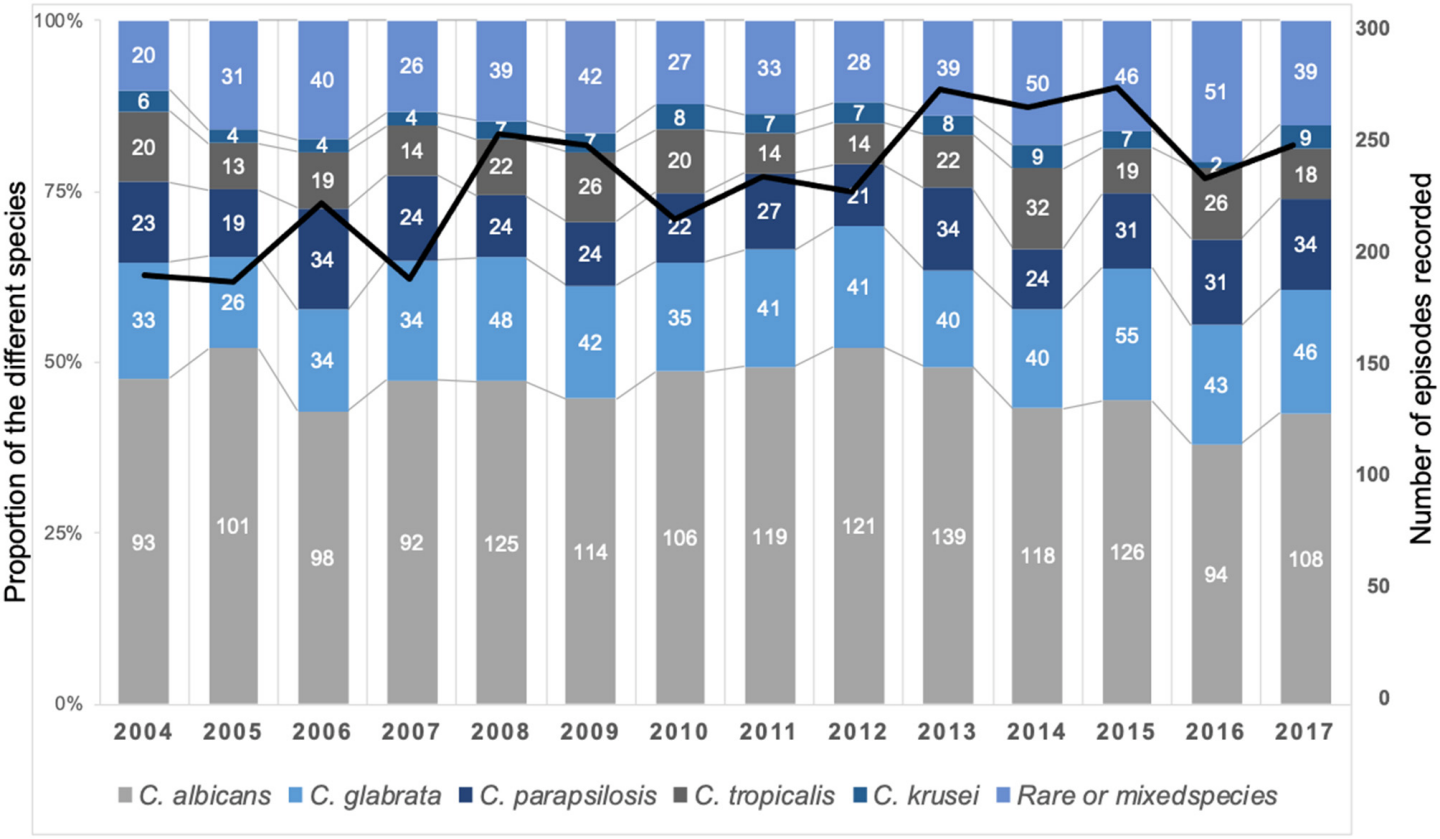
Stable global distribution of species responsible for fungemia during the YEASTS program (Paris area, 2004–2017). Figures in the histograms indicate the number of fungemia due to the five more frequent species, the last category regrouping fungemia due to rare species (<2% of isolates) or to mixed species. A total of 3,363 isolates was recovered during 3,257 episodes in 3,092 patients. The black line represents the evolution of the number of episodes over time (linear regression, *R*^2^ = 0.5101).

A total of 3,363 isolates were recovered with five major species [*Candida albicans* (*n* = 1,614, 48.0%), *Candida glabrata* (*n* = 607, 18.1%), *Candida parapsilosis* (*n* = 390, 11.6%), *Candida tropicalis* (*n* = 299, 8.9%), *C. krusei* (*n* = 96, 2.9%)], and rare species defined as accounting for <2% each (*n* = 357, 10.6%) listed in Supplementary Table 1. Globally, there was no significant evolution in this distribution over time (Figure 2). The distribution of species varied according to the underlying risk factor with more non-*albicans* species in hematology compared to other groups species (*p* < 0.0001) but the distribution did not differ significantly in ICU compared to outside ICU (Supplementary Figure 1). The distribution of species differed significantly between incident (*n* = 3,092) and recurrent episodes (*n* = 165) with less *C. albicans*, more *C. parapsilosis*, and *C. krusei*, and more of rare and mixed species during recurrent episodes (Supplementary Figure 2). The same species in recurrent episode was observed in only 14 cases (8.5%) and increased echinocandin MIC was observed in only two *C. glabrata* isolates (associated with mutation in the *FKS* gene for 1 patient), increased caspofungin MIC in one *Clavispora lusitaniae* (associated with mutation in *FKS* gene) and increased fluconazole MIC in two *C. lusitaniae* isolates.

There was no significant change in the proportion of isolates with fluconazole MIC >32 mg/L or caspofungin MIC >0.25 mg/L (Supplementary Figures 3A,B). Among the 2,661 isolates of common species with natural susceptibility to candins, only 18 isolates (0.68%) had acquired resistance (3 *C. albicans*, 12 *C. glabrata*, 2 *C. krusei*, and 1 *C. tropicalis*) and they were evenly distributed over time (not shown).

### Trends in Drug Prescription

**Figure 3:**
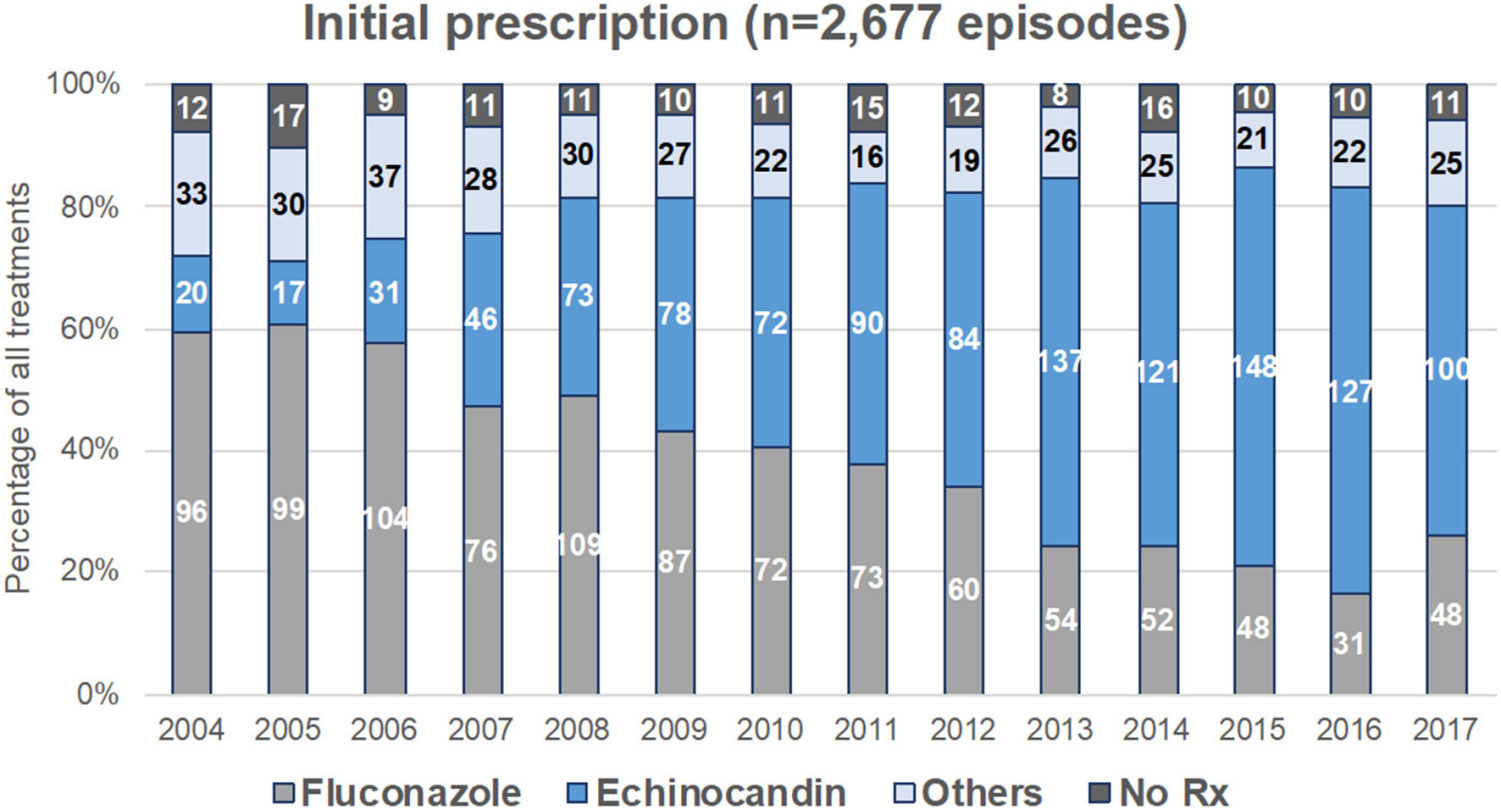
Evolution of first-line antifungal drug prescription for treatment of yeast fungemia (YEAST program, Paris area, 2004–2017). First-line treatment was analyzed for the 2,677 episodes for which time to potential death exceeded 48 h. Figures represent the number of episodes treated each year by fluconazole alone, echinocandin alone, other treatments (liposomal amphotericin B, voriconazole, posaconazole, itraconazole, or drug combination), or not treated.

Drug prescription after blood sampling was analyzed for 2,677 episodes. There was a significant decreased in fluconazole prescription and that of other treatments, and a significant increase in echinocandin prescription over time (*p* < 0.0001, Figure 3), while the proportion of patients receiving no treatment was stable. This was observed both in ICU and outside ICU, and in the three groups studied (Figure 1B). The time to switch from fluconazole to echinocandins differed according to the environment. Outside ICU, it occurred as soon as 2006 in Hematology, 2014 in Oncology, and 2011 in No-malignancy, and for the three groups around 2009–2010 in ICU (Figure 1B). Echinocandins were massively represented by caspofungin since only 39 patients were prescribed micafungin and 10 anidulafungin over the period studied.

**Figure 4:**
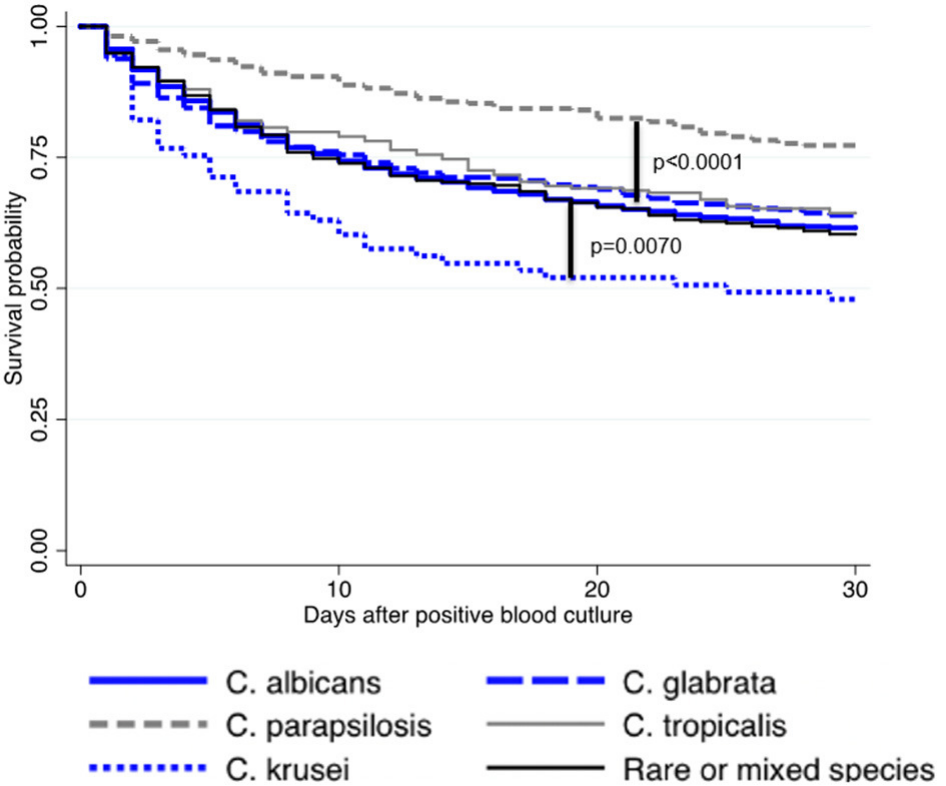
Kaplan–Meier curves illustrating survival rate after incident candidemia according to the species involved in case of single infection due to the five major species or to rare or mixed species (YEASTS program, Paris area, 2004–2017)—(logrank-test *p* < 0.0001). Survival following infection with *C. albicans* (solid blue line), *C. glabrata* (dashed blue line), *C. parapsilosis* (dashed gray line), *C. tropicalis* (solid gray line), rare or mixed species (solid black line) are presented. Compared to *C. albicans*, survival probability was lower with *C. krusei* (*p* = 0.0070) and better with *C. parapsilosis* (*p* < 0.0001).

The effect of initial antifungal drug prescription was assessed on survival after incident episodes of fungemia due to the 5 major *Candida* species or to rare or mixed species. Overall, being infected by *C. krusei* was significantly associated with mortality compared with any other species, and *C. parapsilosis* had the best prognosis (log rank-test *p* < 0.0001, Figure 4). In all cases, the lack of antifungal treatment was significantly associated with mortality except for *C. parapsilosis* fungemia (*p* < 0.001) (not assessable for *C. krusei* for lack of non-treated cases) (Supplementary Figures 4, 5D). For all species, including rare and mixed species but excluding *C. tropicalis*, the choice of the first-line drug had no significant impact on survival (Supplementary Figure 4). For *C. tropicalis*, survival following “other treatment” was improved only when compared to echinocandin alone. Overall, fluconazole alone was associated with similar survival whatever the infecting species (Supplementary Figure 5A, not assessable for *C. krusei* for lack of prescription), whereas survival associated with echinocandin alone (Supplementary Figure 5B) or other prescriptions (Supplementary Figure 5C) differed according to the species involved. Compared to *C. albicans*, survival was significantly better for *C. glabrata* and *C. parapsilosis* fungemia (*p* = 0.0098) following echinocandin treatment (Supplementary Figure 5B), and for fungemia due to *C. parapsilosis* and rare/mixed species following other treatments (Supplementary Figure 5C, *p* = 0.0361). In the multivariate analysis (Table 2), the parameters independently associated with increased death at day 30 were a stay in ICU, an age over 70 years, malignancy as underlying disease (hematology or solid tumor), a diagnosis before 2010, and the absence of antifungal treatment in the first 48 h or, to a lesser extent, the prescription of echinocandin (prescription of fluconazole as reference), whereas an infection by *C. glabrata* or *C. parapsilosis* were protective.

**Table 2:** Independent parameters associated with outcome at 30 days in 2,868 patients diagnosed with incident yeast fungemia, univariate, and multivariate analysis (YEASTS program, Paris area, 2004–2017).

	**Univariate analysis**			**Multivariate analysis**	
	**Death (*****n*** **=** **1,103)**	**Survival (*****n*** **=** **1,765)**	***p***	**OR [95% CI]**	***p***
**Male sex**	60.9% (672)	60.6% (1,070)	0.872		
**Age over 70 years**	37.2 (410)	23.5% (415)	<0.0001	1.72 [1.41–2.10]	<0.0001
**Diagnosis before 2010**	43.0% (474)	39.3 (694)	0.053	1.37 [1.13–1.67]	0.001
**Stay in intensive care unit**	62.6% (690)	37.9% (668)	<0.0001	2.88 [2.36–3.50]	<0.0001
**Main underlying condition**			0.078		
Haematology	19.1% (311)	22.2% (391)		1.47 [1.40–1.89]	<0.0001
Oncology	31.1% (343)	31.8% (562)		1.60 [1.28–1.99]	0.011
No malignancy	49.8% (549)	46.1% (812)		1	
**Species involved**			<0.0001		
*C. albicans*	51.0% (563)	47.1 (832)		1	
*C. glabrata*	17.0% (188)	16.9% (298)		0.71 [0.54–0.92]	0.011
*C. parapsilosis*	6.8% (75)	13.9% (245)		0.54 [0.39–0.75]	<0.0001
*C. tropicalis*	8.4% (93)	8.6% (151)		0.88 [0.63–1.23]	0.455
*C. krusei*	3.8% (42)	2.0% (35)		1.36 [0.77–2.39]	0.291
Rare and mixed species	12.9% (142)	11.6% (204)		1.48 [0.86–1.53]	0.346
**First-line antifungal treatment**			<0.0001		
Fluconazole alone	33.1% (257/776)	40.7% (711/1,749)		1	
Echinocandin alone	40.6% (315/776)	43.2% (755/1,749)		1.26 [1.01–1.58]	0.041
Other drug or combination	11.7% (91/776)	13.6% (237/1,749)		1.06 [0.78–1.45]	0.689
No treatment	14.6% (113/776)	2.6% (46/1,749)		7.85 [5.31–11.60]	<0.0001

Prior exposure to antifungal drugs (fluconazole or echinocandins) was reported in 331/3,092 (10.7%) of incident episodes and 97/165 (59.8%) of recurrent episodes (*p* < 0.001) with no significant change over time (Supplementary Figures 3C,D) except for echinocandins [increase from 1.2% (2/190) in 2004 to 13.5% (23/248) in 2017, *p* = 0.0008], in relation with an increase before incident episode of fungemia (*p* = 0.0071) in ICU (*p* = 0.0001) for patients with no malignancies [from 0 (0/55) to 10.3% (8/78), *p* = 0.0014]. There was no difference in the mortality rate when comparing patients according to prior exposure to antifungal drugs and those without (data not shown).

## Discussion

This present surveillance program within 14 university hospitals shows over the last 14 years (i) a steady mortality rate around 38.2% (51.0% in ICU) for the whole population of patients at day 30 with no improvement over time (ii) a progressive replacement of fluconazole in favor of echinocandins as first-line treatment, and (iii) a stable *Candida* species distribution with no dramatic increase in antifungal resistance.

The global mortality reported here in the YEASTS program is in the same order as recently reported in a meta-analysis of European studies, between 37 and 38% (17). The present sustained mortality rate over time cannot be ascribed to an increase in a specific underlying disease since the figures were unchanged between hematology, oncology or no-malignancy. This could not either be ascribed to a modification in the microbial ecosystem since the species distribution remained globally unchanged and resistance either to fluconazole or echinocandins remained low, and even globally decreased for fluconazole. In contrast, the increasing age of the population (from 48.7% over 60-year of age in 2004 to 56.8% in 2017) plays certainly a role in the sustained high mortality rate (18). In the same way, the proportion of hematology patients in ICU significantly increased, suggesting that fungemia was associated with more severe conditions over time. We also show the constant increase in the mean incidence rate/1,000 admissions from 0.63 to 0.77 (2010–2017) and the incidence rate/10,000 bed days (patient days) from 1.12 to 1.26 (2010–2017) in the 14 hospitals of the survey. These figures are in the same range as those reported in a recent meta-analysis, between 0.96/1,000 admissions in tertiary care centers and 0.52/1,000 in a mixed group of teaching and general hospitals (17). The increase from 0.63 to 0.77 per 1,000 admissions can be due in part to the increase in incident cases but the parallel decrease by 5.6% of hospital beds could have mitigated this explanation. Patients with more severe conditions and thus more prone to fungal infections could have been more often hospitalized. If this hypothesis were true, this might have hampered the improvement of fungemia prognosis expected with the new treatments and recommendations. Unexpectedly, we also found that the prescription of echinocandin was associated with a poorer outcome compared to fluconazole (*p* = 0.041) and this could reflect the clinicians' preference for echinocandins when the patient condition is worsening. Since we could not collect severity markers in the different populations, we cannot assess this hypothesis.

The other major point is the progressive replacement of fluconazole by echinocandins as first line therapy for fungemia. The length of our study allowed covering the launch of caspofungin and micafungin in France and its increasing use over time following the different guidelines (4, 7, 9, 10). The switch fluconazole/echinocandin was almost complete in 2016 although with different timings according to the underlying disease and the ICU context, sooner in hematology and later in oncology outside ICU. This switch is mainly supported by the observed benefit of echinocandin use over azole treatment in the analysis of seven clinical trials by Andes et al. who observed a decreased mortality [OR: 0.65 (95% CI, 0.45–0.94); *P* = 0.02] (11). However, observational studies in France (19, 20), Brasil (21), or Spain (22) have not shown that fluconazole was associated with an excess of mortality. Our observation confirms that the replacement of fluconazole by echinocandin as first line therapy was not associated with the expected improvement of yeast fungemia prognosis. Indeed, as mentioned above, the prescription of echinocandin was associated with a poorer outcome compared to fluconazole (*p* = 0.041). However, this statement warrants to be nuanced since multivariate analysis showed that a diagnosis before 2010 was associated with a poorer prognosis. This could suggest a positive impact of the switch fluconazole/echinocandin after this date, although this improvement over time could also be due to a better global management of fungemia after 2010 or a better prognosis of the underlying disease.

The positive observation made possible by the centralization of isolates and their characterization is the unchanged ecology of major fungal species despite changes in drug prescriptions although we confirmed the different fungal species distribution according to the underlying disease with less *C. albicans* in hematology than in solid cancers and in no-malignancy (23, 24). Thus, we did not observe the emergence of rare species nor did we see the emergence of isolates/species with decreased susceptibility to antifungal drugs, as reported with the SENTRY antifungal Surveillance program (25). In particular, the increase of echinocandin resistance and multidrug resistant candida reported elsewhere was not observed (26–28). More specifically, we did not observe an increase in multidrug resistant *C. glabrata* isolates over 14 years in contrast with other reports (29, 30), nor an increase in fluconazole resistance in *C. parapsilosis* (31). Therefore, if we did observe an overall increase in prior exposure of caspofungin from 1.2% in 2004 to 13.5% in 2017, we did not observe an increase in antifungal MICs or acquired resistance to antifungal drugs in breakthrough fungemia, at least until 2017. Instead, we observed a change in the species distribution between breakthrough fungemia and the other fungemia as already reported (32).

Improving the prognosis of fungemia, still associated with a mortality rate of 38.5% at day 30 in the present study, remains therefore challenging and cannot rely only on the adherence to starting treatment with an echinocandin according to our observation. In reporting a decreased incidence of candidemia, Cleveland et al. argue that the better trends were ascribed to (i) better management of catheters, (ii) use of antifungal prophylaxis, and (iii) improvements in infection control such as hand hygiene (28). Central venous catheters are present in candidemia in the vast majority of cases (here 71.4%) and there is a strong incitation to remove them when feasible (1, 4, 7, 9)), although no randomized clinical trial supports these practices (33). Regarding antifungal drug prophylaxis, its wider use reported by Cleveland et al. was followed by an increase in echinocandin-resistant and multidrug resistant *Candida* (34). Prophylaxis was probably not very common in our cohort, except for the hematological patients for whom prophylaxis targets mold infections. Thus, the number of patients with prior exposure was limited (10.7% of incident episodes and 59.8% of recurrent episodes). Therefore, it is possible that we did not observe emergence of resistance simply because, even if increasing, echinocandin prescription has not reached the level it has in the USA, especially as prophylaxis (35). However, prophylaxis is clearly not advocated in ICU by some authors (36), and even empirical treatment initiated for suspected fungal infection does not modify the fungal infection–free survival at day 28, at least in non-neutropenic critically ill patients with ICU-acquired sepsis (37). Finally, global hygiene control might decrease the incidence and spread of resistant *Candida* isolates as recently underlined with *Candida auris* outbreak control (38). Diagnosis tools could also be improved. Fungal DNA amplification has not shown decisive results (39), whereas T2 magnetic resonance shows promising results (40). The means of improvement could also be different according to the underlying settings, ICU vs. non-ICU, as recently proposed (41).

Our surveillance program suffers from several limitations common to observational studies. Beside the limited geographic coverage, we did not record the number of positive blood cultures, the timing of central venous catheter removal, or potential concomitant mold infections or organ failure which can interfere with the choice of an antifungal drug. Moreover, only the first antifungal prescribed was considered whereas one knows that several lines are often prescribed because of unsatisfactory evolution, side effects, therapeutic interactions, or subsequent results on species identification. All the above parameters could have impacted the prognosis. In some instances, clinicians may also have decided to maintain only palliative cares, which is evidenced here by the poor prognosis of patients not receiving antifungal drugs. As a consequence, the cause of death could not be assessed. However, the lack of difference in survival of fungemia due to specific species whatever the drug prescribed is consistent with the absence of differential effect of the drugs. This is a strong argument for the pre-eminent role of the underlying conditions on survival. This is shown by multivariate analysis on the independent parameters involved for hematology patients when the species is not anymore a parameter influencing death, nor is the initial antifungal drug choice as long as a treatment is prescribed (23). Another independent study on the antifungal therapy according to susceptibility breakpoints on *Candida* spp. concluded that appropriate antifungal treatment was not associated with a better survival (42).

In conclusion, our data show that mortality associated with fungemia-related bloodstream infection has not improved over the last decade. The continuous increase in echinocandin prescription has not been accompanied by an improvement in the mortality rate whatever the underlying disease. The steady mortality rate would be a sign that fungemia occurred in sicker patients rather than a poor efficacy of the current treatments. Although this observation is not *per se* a proof of the no better efficacy of echinocandin over fluconazole, the global care strategy for fungemia seems non-optimal at least in our setting. Therefore, even if new antifungals are urgently needed (43), other trails should be explored such as improvement in catheter management (28), analysis of immune response or inherited patient-specific factors (44), and better diagnostic tools (40).

## Data Availability Statement

The original contributions generated in the study are included in the article/Supplementary Material, further inquiries can be directed to the corresponding author.

## YEASTS Program of The French Mycoses Study Group

The following investigators participated in the YEASTS program of the French Mycosis Study Group (by alphabetical order of the hospital name):

Nawel Ait-Ammar, Anne-Laure Roux, Jean Dunand (hôpital Ambroise Paré, Boulogne); Claire Bouges-Michel, Sophie Brun (hôpital Avicenne, Bobigny); Christian Chochillon, Christine Bonnal (hôpital Bichat, Paris), Françoise Botterel, Adela Angoulvant, Christine Bonnal (hôpital du Kremlin Bicêtre, Kremlin-Bicêtre); André Paugam, Naima Dahane (hôpital Cochin, Paris); Stéphane Bretagne, Françoise Botterel, Nawel Ait-Ammar (hôpital Henri Mondor, Créteil); Elisabeth Chachaty (Institut Gustave Roussy, Villejuif); Isabelle Poilane (hôpital Jean Verdier, Bondy); Claire Lacroix, Stéphane Bretagne, Samia Hamane, Alexandre Alanio (hôpital Lariboisière, Paris); Liliana Mihahila, Najiby Kassis-Chikhany, Adela Angoulvant (hôpital Paul Brousse, Villejuif); Patricia Mariani (hôpital Robert Debré, Paris); Christine Lawrence, Anne-Laure Roux (hôpital Raymond Poincaré, Garches); Claire Lacroix, Alexandre Alanio, Stéphane Bretagne, Blandine Denis, Samia Hamane (hôpital Saint Louis, Paris); Odile Eloy (Centre Hospitalier, Versailles).

## Author Contributions

SB and FD designed the study. KS, MD-O, and the French Mycoses Study Group provided data. SB, MD-O, KS, OL, and FD analyzed the data. SB and FD drafted the first manuscript. SB, MD-O, OL, and FD revised the manuscript. DC analyzed data and revised manuscript. All authors validated the final version of the manuscript.

## Conflict of Interest

SB has received travel grant from Pfizer in 2018 and has served on scientific advisory board for Gilead until 2018. The remaining authors declare that the research was conducted in the absence of any commercial or financial relationships that could be construed as a potential conflict of interest.

## Supplementary Material

Supplementary Figure 1 Uneven distribution of species according to the underlying risk factors and the hospitalization in or outside intensive care unit (ICU) during the YEASTS program (Paris area, 2004–2017, *p* < 0.0001). Figures indicate the number of fungemia due to the five more frequent species, the last category regrouping fungemia due to rare species (<2% of isolates) or to mixed species. A total of 3,363 isolates was recovered during 3,257 episodes in 3,092 patients.

Supplementary Figure 2 Significant change in the species responsible for incident and recurrent episodes of fungemia (*p* = 0.002, YEASTS program, Paris area, 2004–2017).

Supplementary Figure 3 Evolution of antifungal drug resistance and pre-exposure to antifungal drugs over time (YEASTS program, Paris area, 2004–2017). **(A,B)** Evolution of the proportion of isolates with elevated minimal inhibitory concentrations (MIC) of fluconazole **(A)** or caspofungin **(B)** among the 3,363 yeasts responsible for fungemia. Number of resistant isolates per year are indicated in the graphs. For **(A)**, number of isolates with fluconazole MIC > 32 mg/L are determined among all species studied except *C. krusei*. Majority of resistant isolates (75%, 86/114) are *C. glabrata*. For **(B)**, number of isolates with caspofungin MIC > 0.25 mg/L are determined among all species except those intrinsically resistant to echinocandins (*Geotrichum* sp. and basidiomycetous yeasts). Majority of isolates with caspofungin MIC > 0.25 mg/L are *C. parapsilosis* (81%, 149/183), followed by *C. glabrata* (7%, 12/183). **(C,D)** Evolution of the proportion of episodes for which pre-exposure to fluconazole **(C)** and echinocandin **(D)** was recorded among the 3,257 episodes of fungemia. No significant trend was observed outside (dotted line) or inside (solid line) intensive care unit (ICU), except for pre-exposure to echinocandin in ICU (*p* = 0.0001).

Supplementary Figure 4 Kaplan–Meier curves illustrating survival rate after incident candidemia according to the first line antifungal drug prescription in case of single infection due to the five major species or to rare or mixed species (YEASTS program, Paris area, 2004–2017). The total number of patients concerned is provided. Fluconazole (solid blue line), echinocandins (solid thick black line), other drugs or combinations (dash gray line), no treatment (solid thin black line). For *C. krusei*, the small number of patients treated with fluconazole (*n* = 2) and not treated (*n* = 3) prevented their inclusion in the analysis. *P*-value provided after logrank-test.

Supplementary Figure 5 Kaplan–Meier curves illustrating survival rate according to first-line antifungal treatment on survival after incident episode of fungemia due to 5 major species of *Candida* or to rare/mixed species (logrank test). Fluconazole (**A**, 966 patients), candins (**B**, 1,070 patients), other drugs or combinations (**C**, 328 patients), or no antifungal drug (**D**, 159 patients). *Candida albicans* (solid blue line), *C. glabrata* (dash line blue), *C. parapsilosis* (dash gray line)*, C. tropicalis* (solid gray line), *C. krusei* (short dash blue line), and rare or mixed species (thin solid black line). The small number of patients infected by *C. krusei* and treated with fluconazole (*n* = 2) and not treated (*n* = 3) prevented their inclusion in the analysis.

Supplementary Table 1 Number of isolates corresponding to the rare species accounting for <2% of the 3,363 isolates recovered during the YEASTS program (Paris area, 2004–2017).
