# Supplementary material for: No Impact of Fluconazole to Echinocandins Replacement as First-Line Therapy on the Epidemiology of Yeast Fungemia (Hospital-Driven Active Surveillance, 2004–2017, Paris, France)
Source: Front Med (Lausanne). 2021 Apr 20;8:641965. doi: 10.3389/fmed.2021.641965 (PMC8093410; doi:10.3389/fmed.2021.641965)
Supplement: Supplementary Figure 1 — Uneven distribution of species according to the underlying risk factors and the hospitalization in or outside intensive care unit (ICU) during the YEASTS program (Paris area, 2004–2017, p < 0.0001). Figures indicate the number of fungemia due to the five more frequent species, the last category regrouping fungemia due to rare species (<2% of isolates) or to mixed species. A total of 3,363 isolates was recovered during 3,257 episodes in 3,092 patients. [file Data_Sheet_1.PDF]

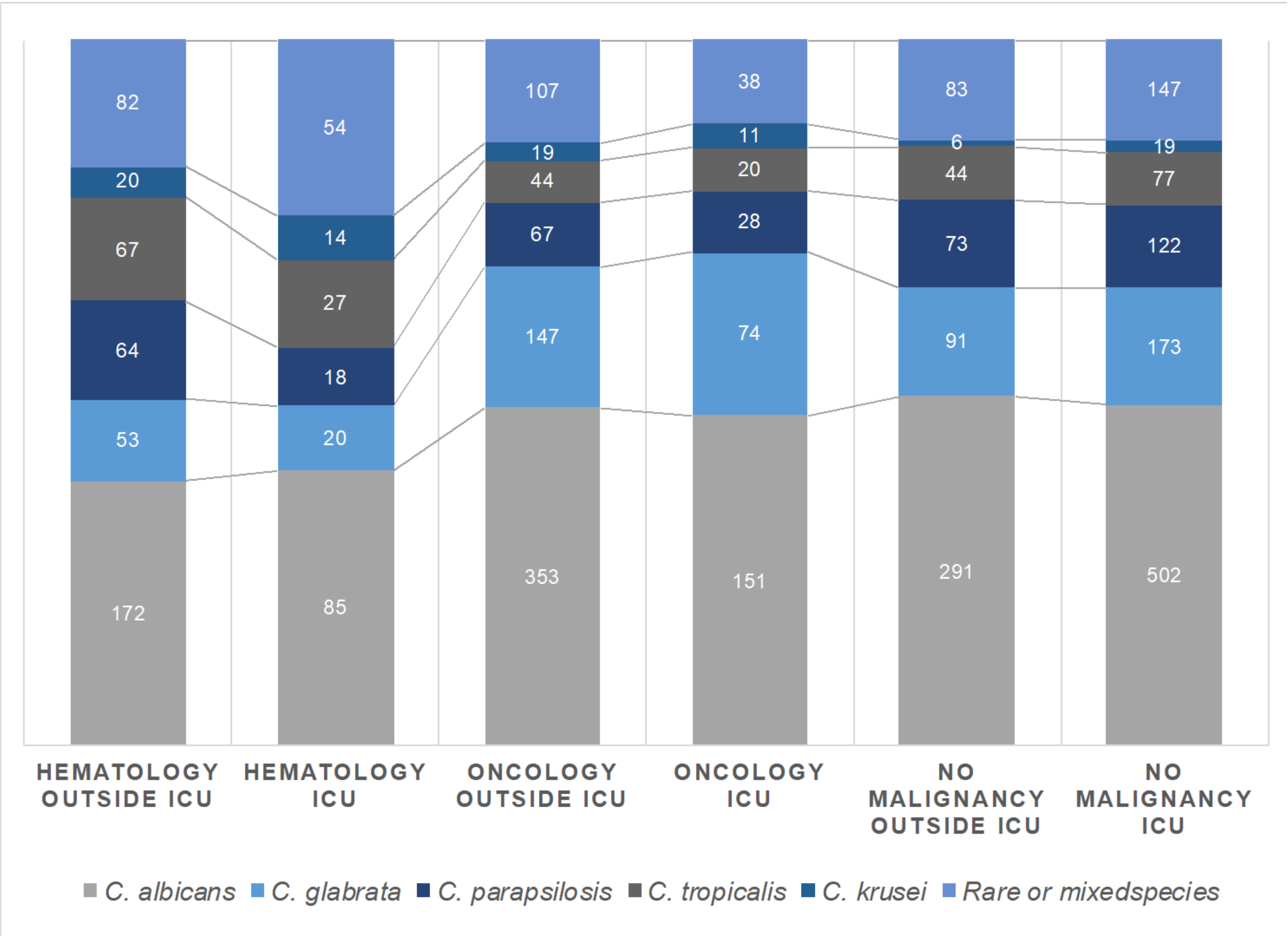

Supplemental Figure 1:

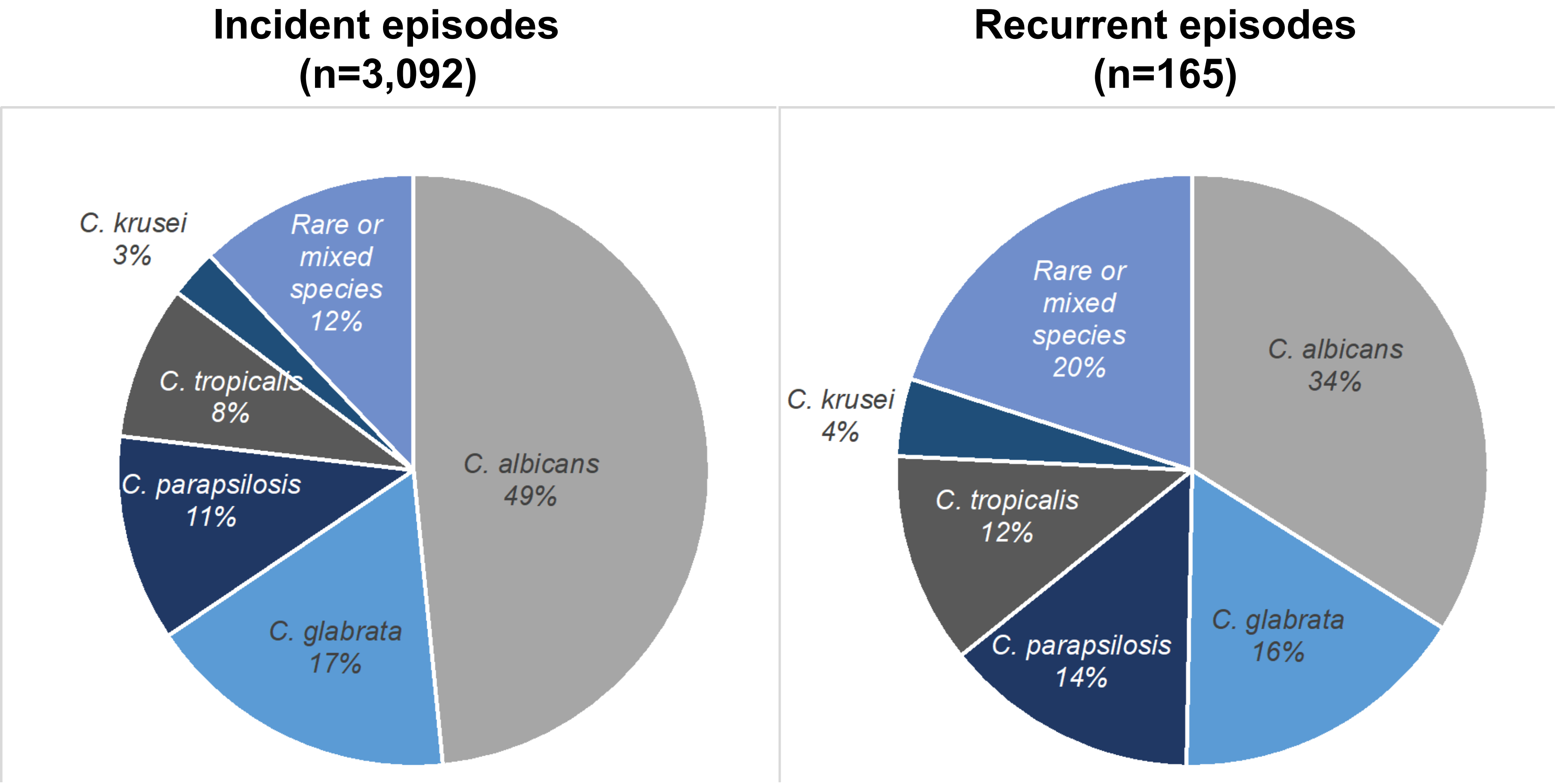

Supplemental Figure 2

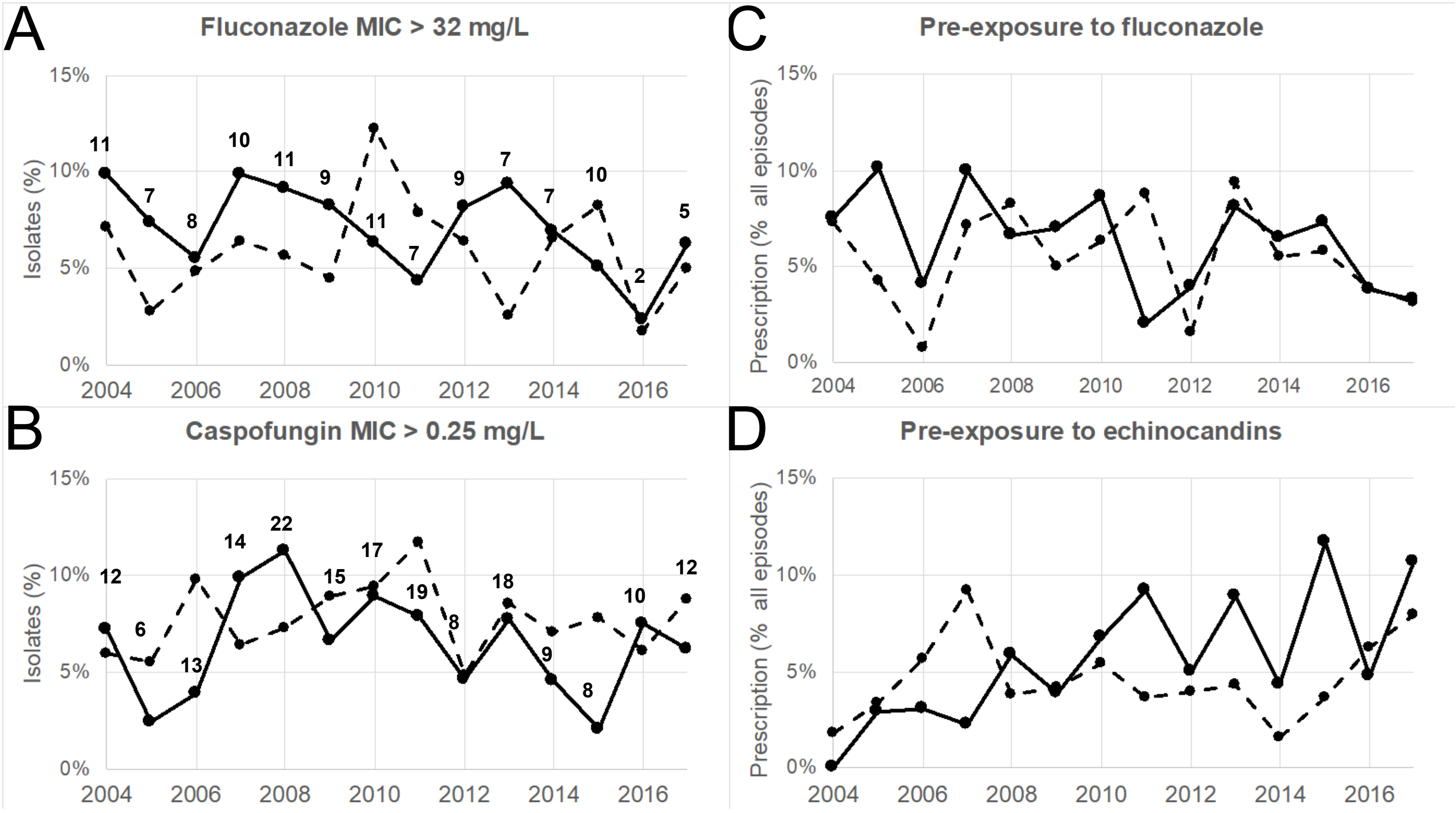

Supplemental Figure 3

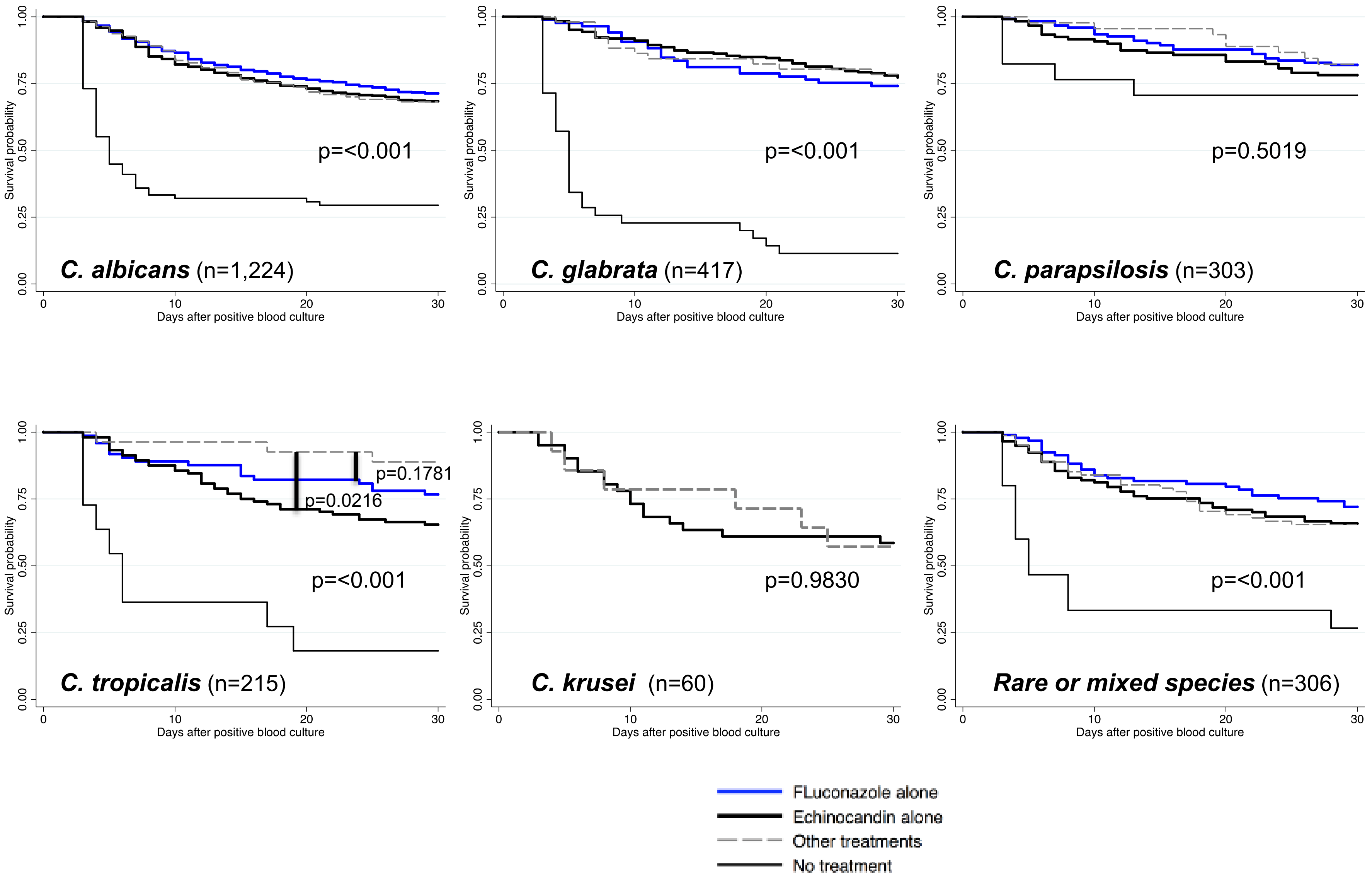

Supplemental Figure 4

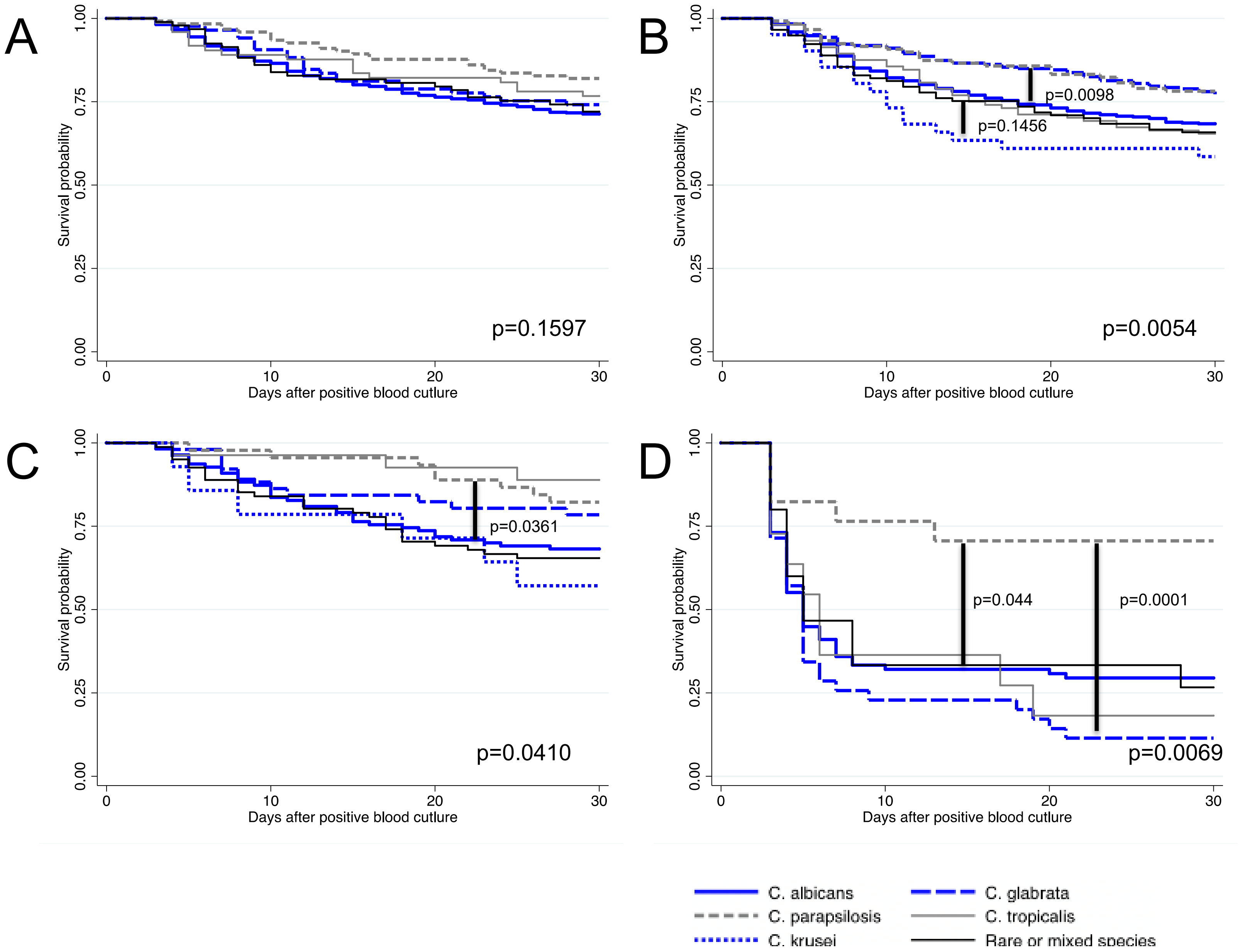

Supplemental Figure 5

Table S1: Number of isolates corresponding to the rare species accounting for less than 2% of the 3,363 isolates recovered during the YEASTS program (Paris area, 2004-2017)

	Denomination	No. of isolates	%
**Ascomycetes**			
	Clavispora lusitaniae (Candida lusitaniae)	62	1.84%
	Kluyveromyces marxianus var marxianus (Candida kefyr)	58	1.72%
	Candida dubliniensis	54	1.61%
	Meyerozyma guilliermondii (Candida guilliermondii)	25	0.74%
	Candida metapsilosis	17	0.51%
	Candida orthopsilosis	13	0.39%
	Candida inconspicua	10	0.30%
	Cyberlindnera jadinii (Candida utilis)	10	0.30%
	Wickerhamomyces anomalus (Candida pelliculosa)	9	0.27%
	Meyerozyma caribbica (Candida fermentati)	5	0.15%
	Candida rugosa	3	0.09%
	Kodamaea ohmeri (Pichia ohmeri)	3	0.09%
	Cyberlindnera fabianii	3	0.09%
	Candida haemulonii	2	0.06%
	Candida duobushaemulonii	2	0.06%
	Candida pseudohaemulonii	1	0.03%
	Candida nivariensis	2	0.06%
	Pichia norvegensis	2	0.06%
	Candida homilentoma	1	0.03%
	Candida palmioleophila	1	0.03%
	Candida pararugosa	1	0.03%
	Kuyveromyces lactis var. lactis (Candida sphaerica)	1	0.03%
	Yarrowia lipolytica (Candida lipolytica)	2	0.06%
	Saprochaete clavata (Geotrichum clavatum)	13	0.39%
	Magnusiomyces capitatus (Geotrichum capitatum)	10	0.30%
	Saccharomyces cerevisiae	14	0.42%
**Basidiomycetes**			
	Rhodotorula mucilaginosa	9	0.27%
	Trichosporon asahii	11	0.33%
	Trichosporon inkin	2	0.06%
	Trichosporon sp.	8	0.24%
	Malassezia furfur	1	0.03%
	Malassezia pachydermatis	1	0.03%
